# The Impact of Rurality and Disadvantage on the Diagnostic Interval for Breast Cancer in a Large Population-Based Study of 3202 Women in Queensland, Australia

**DOI:** 10.3390/ijerph13111156

**Published:** 2016-11-19

**Authors:** Philippa H. Youl, Joanne F. Aitken, Gavin Turrell, Suzanne K. Chambers, Jeffrey Dunn, Christopher Pyke, Peter D. Baade

**Affiliations:** 1 Cancer Council Queensland, P.O. Box 201, Spring Hill, QLD 4004, Australia; joanneaitken@cancerqld.org.au (J.F.A.); suzanne.chambers@griffith.edu.au (S.K.C.); jeffdunn@cancerqld.org.au (J.D.); peterbaade@cancerqld.org.au (P.D.B.); 2 Menzies Health Institute Queensland, Griffith University, Gold Coast, QLD 4222, Australia; 3 School of Public Health and Social Work, Queensland University of Technology, Herston Road, Kelvin Grove, QLD 4059, Australia; 4 School of Population Health, University of Queensland, Brisbane, QLD 4072, Australia; 5 Institute for Resilient Regions, University of Southern Queensland, Toowoomba, QLD 4350, Australia; 6 Institute of Health and Ageing, Australian Catholic University, Fitzroy, VIC 3115, Australia; Gavin.Turrell@acu.edu.au; 7 School of Social Science, University of Queensland, Brisbane, QLD4072, Australia; 8 Mater Medical Centre, 293 Vulture Street, South Brisbane, QLD 4101, Australia; c_pyke@mc.mater.org.au; 9 School of Mathematical Sciences, Queensland University of Technology, Gardens Point, Brisbane, QLD 4000, Australia

**Keywords:** breast cancer, delay, diagnosis, rurality, inequalities, health system

## Abstract

Delays in diagnosing breast cancer (BC) can lead to poorer outcomes. We investigated factors related to the diagnostic interval in a population-based cohort of 3202 women diagnosed with BC in Queensland, Australia. Interviews ascertained method of detection and dates of medical/procedural appointments, and clinical information was obtained from medical records. Time intervals were calculated from self-recognition of symptoms (symptom-detected) or mammogram (screen-detected) to diagnosis (diagnostic interval (DI)). The cohort included 1560 women with symptom-detected and 1642 with screen-detected BC. Symptom-detected women had higher odds of DI of >60 days if they were Indigenous (OR = 3.12, 95% CI = 1.40, 6.98); lived in outer regional (OR = 1.50, 95% CI = 1.09, 2.06) or remote locations (OR = 2.46, 95% CI = 1.39, 4.38); or presented with a “non-lump” symptom (OR = 1.84, 95% CI = 1.43, 2.36). For screen-detected BC, women who were Indigenous (OR = 2.36, 95% CI = 1.03, 5.80); lived in remote locations (OR = 2.35, 95% CI = 1.24, 4.44); or disadvantaged areas (OR = 1.69, 95% CI = 1.17, 2.43) and attended a public screening facility (OR = 2.10, 95% CI = 1.40, 3.17) had higher odds of DI > 30 days. Our study indicates a disadvantage in terms of DI for rural, disadvantaged and Indigenous women. Difficulties in accessing primary care and diagnostic services are evident. There is a need to identify and implement an efficient and effective model of care to minimize avoidable longer diagnostic intervals.

## 1. Introduction

An estimated 1.67 million women were diagnosed with breast cancer worldwide in 2012 and rates are continuing to rise [1]. In Australia, breast cancer is the most common cancer diagnosed amongst women (with the exception of keratinocyte cancer) and the second leading cause of cancer death [2]. Incidence and mortality rates are similar to those observed in other developed countries [1].

Detection of breast cancer at an early stage is associated with lower mortality and improved survival [3]. Observed five-year survival varies from 88% for stage I breast cancers to 15% for stage IV [4]. The goal is always to diagnose cancers at the earliest possible stage providing patients with the best possible prognosis. The majority of adult cancers are slow growing, with one study suggesting breast cancers were diagnosed, on average, approximately seven years after the initial onset [5]. However, this is dependent on a number of factors including tumour grade, rate of growth and morphological type and likely other unknown factors [5]. Despite this variability, there is consensus in the literature that longer intervals between first noticing a breast cancer symptom and definitive diagnosis are associated with larger tumour size and later stage tumours [6,7,8,9,10]. Further, it has been established that intervals of greater than three to six months are associated with poorer survival [6,11]. While intervals of a few days or a few weeks may not have significant impact on survival, longer intervals in obtaining a diagnosis can mean more stress for a patient [12], and potentially more rigorous treatment regimens and their associated side effects and morbidities for patients whose cancer proves to be more advanced.

Timely detection and diagnosis of breast cancer requires a good awareness of the symptoms on the part of women and doctors, an efficient and effective population-based screening program (for women within the target age-range) and timely access to diagnostic services. However, inequalities in each of these components are evident. In Australia, persistent inequalities in access to breast cancer screening and diagnostic services have been documented. For example, rates of breast screening within the BreastScreen Australia services (Australia’s publically-funded screening program for women aged 50 to 74 years) are lowest for women living in remote and very remote areas of Australia and for Indigenous women [13]. Further, inequalities in timely access to diagnostic services have been reported in Australia [14], and elsewhere [15,16,17].

While in Australia there are published General Practitioner (GP) guidelines for the investigation of a new breast symptom [18], these do not include a recommended time period from initial presentation to commencing relevant diagnostic procedures. However, in the United Kingdom, recommendations state patients should receive an appointment or be seen by a specialist within 14 days of the initial GP consultation, and women presenting with symptoms of invasive cancer should receive a diagnosis within five working days of the initial specialist assessment [19]. In relation to screen-detected breast cancer, there is no international consensus on the appropriate time interval for assessment following an abnormal mammogram. BreastScreen Australia accreditation standards state that 90% of all screened women should receive notification of their mammogram results within 14 days and 100% within 28 days [20]. The American College of Radiology recommends women should be contacted regarding an abnormal mammography within five days [21]. Currently, only the European community have published guidelines that include the timeliness of follow-up diagnostic procedures recommending this should begin within five days of notification of an abnormal mammogram [22].

Various definitions have been applied to “diagnostic delay” or “diagnostic interval”, taking the time between first symptom or a positive screening test and either the histological diagnosis [9,23,24,25], or the commencement of treatment [6,7,26]. For symptomatic breast cancer, diagnostic intervals can be impacted by “patient-related intervals” includes the time from first noticing a symptom to first medical appointment, and/or “system-related intervals”, i.e., the time from first medical appointment to histological diagnosis.

An earlier systematic review published in 1999 found studies examining patient delay by and large, relied on small sample sizes, the non-standardised collection of secondary data, for example medical records, and did not include the underlying reasons why a patient may delay seeking medical attention [27]. While some recent studies have addressed some of the previously identified weaknesses, such as addressing the psychosocial factors that may predict risk of delay [8,28], others have focused on subgroups (such as young or low income women, early breast cancer only) [23,24,29], or have not included reasons for delay [26,30,31]. It has been suggested that the patient-related interval may be influenced by a number of factors including: “demographic factors” (such as age, education); “psychological factors” (such as fear and anxiety); “social factors” (such as work and family commitments); “behavioural factors” (such as symptom recognition, awareness and watchful waiting); and “system factors” (such as access, difficulties in making appointments, distance to medical services, coordination of diagnostic tests, and inadequate communication between and within medical services) [32]. Exclusion of the underlying reasons for longer patient intervals or other potentially important covariates, limits our ability to intervene to reduce the patient- and system-related intervals.

Australia is a geographically large country where diagnostic and treatment services are concentrated in major cities. To our knowledge, there have been no large-scale studies conducted in an Australian population examining the extent to which patient and system factors impact diagnostic intervals. To address this issue we undertook a large population-based longitudinal study of women newly diagnosed with breast cancer in Queensland, to identify predictors of overall diagnostic delay, and patient- and system-related intervals, and to examine reasons for longer intervals.

## 2. Materials and Methods

The Breast Cancer Outcomes study (BCOS) is a longitudinal study among women aged 20 to 79 years newly diagnosed with invasive breast cancer in Queensland, Australia [33].

### 2.1. Ethics

Ethical approval for this study was obtained from the Human Research Ethics Committee of Griffith University, Australia (PSY/C4/09/HREC). Approval to access confidential health information was obtained from the Research Ethics and Governance Unit, Queensland Health.

### 2.2. Study Population

All female residents of Queensland, Australia with a histologically confirmed first primary invasive breast cancer diagnosed between 1 January 2011 and 30 June 2013, aged 20 to 79 years, and notified to the Queensland Cancer Registry were eligible for the study. Cancer notifications have been a legal requirement in Queensland since 1982. Eligible women were required to speak and understand English and have no cognitive impairment that would preclude them participating in a telephone interview.

### 2.3. Recruitment

Letters explaining the study and seeking permission to contact women were sent to all treating doctors. A reminder letter was sent to non-responding doctors approximately two weeks later followed by a telephone call after a further two weeks. Women for whom doctor’s permission was obtained were invited by letter to participate. Non-responders were re-mailed two weeks after the initial mailout and followed by a telephone call after a further two weeks.

We identified 5426 women of whom 66 were deceased. Of the remaining 5360 women, doctor consent was not obtained for 688 (12.8%). The most common reasons doctors cited for refusing consent were their patient was too ill or they did not believe their patient would manage or be interested in participating in the study. Of those with doctor consent (*n* = 4762), consent was received from a total of 3480 women (74.4%) and 3326 eligible women completed the interview (71.2%). There were no significant differences between participants and non-participants in relation to age (*p* = 0.33), however women living in major cities were less likely to participate (*p* = 0.04), and non-participants were more likely to be diagnosed with advanced disease (*p* = 0.03).

As the purpose of this study was to examine diagnostic intervals, including the patient and system components, we excluded 124 women whose breast cancer was initially detected by their doctor (through a clinical breast examination or incidentally). Thus, the final cohort consisted of 3202 participants.

### 2.4. Data Collection

Full details of data collection have been described previously [33]. A validated, semi-structured telephone interview administered by trained health interviewers was used to construct each woman’s pathway to their breast cancer diagnosis. Two groups were defined; symptom-detected and screen-detected. The symptom-detected group included women who first noticed a sign or symptom of breast cancer or where a layperson initially detected a sign or symptom suspicious of breast cancer. The screen-detected group represented women where the suspicion of breast cancer was made on routine mammography or ultrasound.

To ascertain how the breast cancer was detected, participants were asked “How was your breast cancer first detected, and by that I mean who was the very first person who thought or noticed something might be wrong?” If a participant indicated her breast cancer was detected during a screening examination, the interviewer probed further to ensure there was no suspicion of breast cancer prior to the screening examination.

Women were asked for the date they first noticed the symptoms or when the screening test was conducted, the date they first consulted a doctor, the outcome of the appointment (given all clear, wait and monitor, referred, ordered tests), type and date of subsequent procedures and where longer intervals occurred, the reasons for the delay up to the date of confirmed diagnosis. To maximise accuracy, women were provided with the relevant questions prior to their interview and asked to document their responses after consulting their diaries/calendars and/or their doctors. Interviewers used various probes to help with recall as have been used in other studies [26,34,35]. In a random sample of 500 women, the accuracy of self-reported dates of screening and medical appointments was found to be high when checked against medical records. For example, in screen-detected cases (*n* = 290), 85% reported a date within two days of the true date of screening, and for symptom-detected cases (*n* = 210), 70% reported a date of initial medical appointment within a few days of the correct date. While accuracy was slightly lower for older women, there were no significant differences in accuracy according to remoteness or area-level disadvantage.

We collected sociodemographic information pertaining to age, education, marital status, employment, health insurance status, gross household income and residential location. Clinical coders extracted relevant data items from histopathology reports including date of diagnosis, tumour site and size, morphology, histological grade, degree of lymph node involvement, oestrogen, progesterone and Herceptin status, and stage was assigned according to the American Joint Committee Cancer Staging Manual [36]. Tumour size was determined as the diameter of the invasive component of the tumour, or in the case of multicentric tumours, the largest tumour diameter was used.

Remoteness of residence at diagnosis was categorized using the ARIA+ classification, which is a measure of accessibility and remoteness based on geographical location [37]. Area-level socioeconomic disadvantage was measured using the Index of Relative Socioeconomic Disadvantage (IRSD) 2011 version calculated by the Australian Bureau of Statistics (ABS) (Canberra, Australia) [38].

Mean time from diagnosis to interview was 6.8 months (range = 3.4. to 18.5 months) with over 80% of the study cohort being interviewed within 10 months of diagnosis. On average the baseline interview took 60 min (range 15 to 90 min).

### 2.5. Study Outcomes

Diagnostic interval (DI) was defined as the number of days between first detecting a sign or symptom (for the symptom-detected group), or date of mammogram and/or ultrasound (for screen-detected group) to the date of a biopsy proven breast cancer.

For the symptom-detected group, DI was further subdivided into patient-related interval (PI) defined as the number of days between first noticing the symptom to date of first medical appointment and system-related interval (SI), defined as the time from first medical appointment to diagnosis. While there is some overlap between the patient and system components, these definitions focus on who was initiating the action. Participants were additionally asked the reasons for the delay (multiple responses allowed). As suggested by others [32,39], reasons for either PI or SI were then collapsed into four main themes: *competing priorities of the patient* (including work or home/family priorities, other medical problems); *awareness and symptom recognition* (including symptoms not important, had been monitoring the symptoms, decided to wait for regular appointment); *fear and anxiety* (including was worried what might be found, did not like going to doctors); and *system related factors* (including difficulty in obtaining an appointment, distance or difficulty in getting to the doctor, costs).

For screen-detected breast cancers, while other studies have included a definition for PI as the time from mammogram (and/or ultrasound) appointment to first medical procedure [26], this does not take into account the time from mammogram to notification of results which can be impacted by both patient and system factors (screening facility tried to contact patient; notified of results but took some time to contact doctor for follow-up; time taken to view and report on mammogram, referrals from screening facility to patient’s GP or specialist). Thus we defined DI for screen-detected cases as the time from mammogram to confirmatory biopsy. We further examined time from mammogram to receipt of results separately.

### 2.6. Predictors of Delay

We examined the impact of various patient, clinical and health system factors for DI for both groups. These factors included age at diagnosis; Indigenous status; education; employment; gross household income; private health insurance; family history of breast cancer; comorbidities; history of benign breast disease; history of breast self-examination and mammography; whether participant had discussed with a partner/family/friend their concerns about a symptom of breast cancer (for symptom-detected); degree of remoteness; area-level disadvantage; tumour size and grade; and nodal status. We additionally examined these same factors in relation to PI and SI for women with symptom-detected breast cancers.

### 2.7. Statistical Analysis

The statistical significance of bivariate comparisons between DI, PI and SI and various sociodemographic and clinical factors for symptom- and screen-detected breast cancers was estimated using the chi-square test. As the distribution of days was highly skewed we have reported median values along with 25th and 75th percentiles for DI, PI and SI.

We used separate logistic regression models to examine factors independently associated with DI according to detection method. For symptom-detected breast cancers the outcome variable for DI was >60 days vs. ≤60 days which was selected based on previously published work [28], with the outcome variable for both PI and SI being >30 days vs. ≤30 days. For screen-detected breast cancer, DI delay was defined as >30 days vs. ≤30 days. The shorter time interval for screen-detected breast cancers was selected based on the literature recommending time from abnormal mammogram to diagnostic procedures should be within approximately 30 days [22]. The outcome variable for time from mammogram to receipt of results was defined as >14 days vs. ≤14 days, based on BreastScreen Australia’s accreditation standards [20].

For each model, we began with a full logistic model including all variables relating to either symptom- or screen-detected breast cancer along with the clinical characteristics of the breast cancer. Variables not associated with the outcome were then excluded manually on a one-by-one basis using a significance level of >0.20, the model re-run and the likelihood ratio (LR) test was examined at each stage of the variable exclusion process. Excluded variables were then re-introduced into subsequent models and the LR test re-examined to ensure variables associated with the outcome were not excluded. As individuals living in remote and very remote areas are also more likely to live in a socioeconomically disadvantaged area [40], we added an interaction term to examine if associations between DI and area-level disadvantage was different across various levels of remoteness. All analyses were conducted using Stata14.0. (Stata Corp, College Station, TX, USA).

## 3. Results

### 3.1. Description of the Cohort

The cohort included 1560 women with symptom-detected and 1642 women with screen-detected breast cancer. Mean age was 57 years (54 years for symptom- and 60 years for screen-detected). Just under half were diagnosed with stage I breast cancer (48.6%), 38.8% with stage II, 11.1% with stage III/IV and the remaining 1.6% of women were unable to be staged. Just over half (55.1%) lived in major cities, 24.0% in inner regional areas and the remainder (20.9%) lived in outer regional or remote/very remote areas. Nearly two-thirds reported they had full private health insurance (63.5%), 10.4% had some health insurance and the remainder (26.1%) had no health insurance (data not shown).

### 3.2. Symptom-Detected Breast Cancer

Median time to diagnosis from recognition of own symptoms was 25 days (25th percentile = 11 days and 75th percentile = 63 days). Just over a quarter (26.5%) experienced a delay of >60 days and 18% experienced delays of >90 days. Table 1 shows the proportion of women with symptom-detected breast cancer where the DI was >60 days according to sociodemographic, screening history and clinical characteristics of the breast cancer. The proportion of women with DI > 60 days was higher for Indigenous women (*p* = 0.006), women in outer regional and remote/very remote areas (*p* = 0.003), those whose tumour was classified as low grade (*p* < 0.001), women who did not indicate a breast lump was at least one of her symptoms (*p* < 0.001), and for women who had not discussed their symptom with someone close to them prior to their initial medical appointment (*p* = 0.05).

#### 3.2.1. Factors Associated with Diagnostic Delay

Table 1 presents the results of the multivariable logistic regression model for symptom-detected breast cancers including all factors found to be significantly associated with the diagnostic interval. The odds of a DI of >60 days were higher women who were Indigenous (OR = 3.12, 95% CI = 1.40, 6.98) compared to non-Indigenous; lived in an outer regional (OR = 1.50, 95% CI = 1.09, 2.06) or remote/very remote locations (OR = 2.46, 95% CI = 1.39, 4.38) compared to those in major cities. Other independent predictors of a longer DI included the absence of a breast lump as a presenting symptom (OR = 1.84, 95% CI = 1.43, 2.36). Tumour size was not associated with DI, however women whose tumours were high compared to low grade had a lower odds of a longer DI (OR = 0.49, 95% CI = 0.33–0.73).

#### 3.2.2. Patient-Related Interval

Of the 1560 women with symptom-detected breast cancer, 54.7% presented to their medical practitioner within 14 days of symptom discovery, 71.6% within 30 days and 85.3% within 60 days. In the multivariable logistic regression model (Table 2), factors independently associated with a longer PI included the absence of a breast lump as a presenting symptom (OR = 1.88, 95% CI = 1.47, 2.39) and not discussing symptom discovery with someone other than a doctor (OR = 1.48, 95% CI = 1.16, 1.88). Further the odds of a longer PI was higher for women who had no or infrequent mammograms compared to those reporting regular mammography (OR = 1.35, 95% CI = 1.06–1.73), with the direction and strength of this association remaining when we restricted the analysis to women aged 50 to 69 years. Age group, location of residence, area-level disadvantage, education, employment and private health insurance were not independently associated with PI. These results were similar when we defined PI as more than 60 or 90 days.

For women who indicated a delay in seeing a medical practitioner or in making an appointment at a screening facility (*n* = 130), the most common reasons for delay were: lack of awareness of possible symptoms of breast cancer (47.9%); work or family priorities (39.2%); system difficulties (i.e., obtaining appointments or distance to doctor) (26.2%); and fear or anxiety for what might be found (4.9%). A significantly higher proportion of women in regional and remote/very remote locations cited delays in obtaining medical appointments or difficulties in travel time to their doctor (33.9% and 19.2%, respectively, *p* < 0.001). Additionally, women with lower levels of education were more likely to indicate they did not think the symptom was important or it would go away compared to those with post-high school qualifications (53.7% and 44.3%, respectively, *p* = 0.01) (data not shown).

#### 3.2.3. System Delay

Overall, 38.1% of participants were diagnosed within seven days of their initial medical consultation, 62.2% within 14 days and 84.3% within 30 days (the vast majority of women saw a GP for their initial consultation). In the multivariable logistic regression model, where SI was defined as delays of >30 days (Table 3), women living in outer regional areas compared to major cities, and women in areas of disadvantage had a higher odds of a longer SI (OR = 1.61, 95% CI = 1.10, 2.35 and OR = 1.42, 95% CI = 1.02, 2.12, respectively). Tests for an interaction between remoteness and area-level disadvantage and SI were not significant (*p* = 0.45). Compared to women who were retired, women employed full-time were nearly three times more likely to experience a longer SI (OR = 2.87, 95% CI = 1.72, 4.81) while those with larger and high grade tumours had a lower odds of SI, (Table 3).

Participants reported various combinations of diagnostic procedures were conducted with the most common pathway following initial medical consultation being a clinical breast examination, mammogram, ultrasound and biopsy. Just under a quarter cited a delay (defined as 14 days or more) with their mammogram (23.5%), 25.9% a delay with an ultrasound and 31.7% experienced delay with their biopsy. The most common reason for delays was difficulties in obtaining or coordinating appointments for the procedures. For example, for participants who had a delay in their biopsy, 81.3% indicated this was due to difficulties in obtaining an appointment and this was significantly more common for women in regional and remote/very remote areas than for those in major cities (85.6% vs. 70.0%, *p* = 0.03).

### 3.3. Screen-Detected Breast Cancer

#### 3.3.1. Interval from Mammogram to Receipt of Results

For women whose breast cancer was screen-detected (*n* = 1642), median time to receive abnormal mammogram results was seven days with most women notified within 14 days (80.5%). Table 4 presents the results of a fully adjusted logistic regression model examining factors associated with an interval of more than 14 days in receipt of mammogram results. The odds of a longer interval were higher for women in outer regional and remote areas (OR = 4.17, 95% CI = 2.95, 5.91 and OR = 3.62, 95% CI = 2.00, 6.55, respectively) compared to those in major cities and for women who had their mammogram in a public facility (BreastScreen or public hospital) (OR = 9.51, 95% CI = 5.21, 17.36) compared to private facilities. Further, women with tumours ≥1 cm had a lower odds of delay in receipt of results than women with tumours of less than 1 cm (*p* = 0.01).

#### 3.3.2. Diagnostic Delay

The median time between abnormal mammogram and diagnosis was 14 days. While only a minority of women (16.4%) had a diagnostic interval more than 30 days, some groups were more likely to experience intervals beyond 30 days (Table 5). At a bivariate level (percentages shown in Table 5), women who were Indigenous (*p* < 0.001); lived in outer regional and remote locations (*p* < 0.001) or disadvantaged areas (*p* < 0.001); those without private health insurance (*p* = 0.007); and where the screening facility was public (*p* < 0.001) were all more likely to experience a DI greater than 30 days. No significant bivariate associations were found for any other sociodemographic variables.

After adjustment, the odds of experiencing a longer DI for women with screen-detected breast cancer (Table 5) were higher for Indigenous women (OR = 2.36, 95% CI = 1.03, 5.80) than non-Indigenous women, and women from outer regional (OR = 3.32, 95% CI = 2.31, 4.75) and remote locations (OR = 2.35, 95% CI = 1.24, 4.44) compared to those in major cities. Additionally, compared to women from more socioeconomically advantaged areas, women from areas of middle socioeconomic status and those from areas of disadvantage had a higher odds of a longer DI (OR = 1.58, 95% CI = 1.03, 2.56 and OR = 1.69, 95% CI = 1.17, 2.43, respectively). Further, the odds of a longer DI was significantly higher for women whose mammogram was conducted in a public screening facility compared to those attending a private facility (OR = 2.10, 95% CI = 1.40, 3.17).

#### 3.3.3. Reasons for Delay

Most common reasons women cited for a delay from their mammogram to diagnosis were again difficulties in obtaining or coordinating appointments for ultrasounds and biopsies (67.4%), along with work or family commitments (38.4%).

## 4. Discussion

This study examined diagnostic intervals in a large cohort of women with either symptom- or screen-detected breast cancer. For symptom-detected breast cancer median interval between symptom and diagnosis was 25 days, while for screen-detected breast cancer, the median interval from screen to diagnosis was 14 days. These intervals are not excessive from the viewpoint of breast cancer biology [5], however, from the patient viewpoint, waiting for confirmation of a diagnosis is an anxious and stressful time [12,41]. In this study, while we found the majority of symptom-detected breast cancer was diagnosed within 90 days, it remained that one in five women experienced a delay of greater than 90 days, the time at which survival can be impacted [11,42]. For example, Richards et al. [11] found women with delays of greater than 12 week had 12% lower five-year survival compared to those with shorter (<12 weeks) intervals. For both symptom- and screen-detected breast cancer, we found women living in more geographically isolated areas, areas of disadvantage and Indigenous women were more likely to experience longer diagnostic intervals compared with women from major cities, more socioeconomically advantaged areas and non-Indigenous women. We identified a number of factors associated with longer patient and system intervals.

### 4.1. Symptom-Detected Breast Cancer

In this study while the majority of women were diagnosed within 60 days, just over a quarter (26.5%) experienced a diagnostic interval of more than 60 days and 18% experienced intervals of more than 90 days. Two systematic reviews including one meta-analysis, identified delays of 90 days or more were associated with later stage breast cancer and poorer survival [11,42]. While in our study we have not conducted a survival analysis, we did find women who experienced a DI of more than 60 days were significantly more likely to be diagnosed with stage III or IV disease with this association remaining after adjustment for tumour grade.

*Patient-related interval:* The time from symptom discovery to diagnosis includes periods where patient and/or system (medical) factors can impact overall time to diagnosis. Amongst our cohort, the median time from breast cancer symptom to initial medical consultation was 11 days. This is similar to that observed in more recent breast cancer cohorts in the United Kingdom (median of 13 days) [43], New Zealand (median of 14 days) [44], and Germany (median of 16 days) [8]. Our results also add to the growing trend for shorter patient delays to that observed in earlier studies [45,46]. However, it remained in our study that one in seven women waited more than 60 days and one in ten women waited more than 90 days to consult their doctor.

A longer patient interval was not associated with age, geographical location or with most other sociodemographic factors. While Indigenous women were more likely to experience longer overall diagnostic intervals (mainly driven by system delay), they were no more or less likely than their non-Indigenous counterparts to delay initial presentation to their doctor. These results are similar to that observed in New Zealand amongst Maori and Pacific Islander women [44]. However, delays in patient presentation have been reported for a number of ethnicities including Asian, Middle Eastern and African Americans [9,25,47,48,49].

The strongest risk factor for a longer patient interval in this study was the lack of a breast lump as a symptom, with this nearly doubling the risk of delayed presentation. While the majority of women with symptom-detected breast cancer noticed a breast lump (72%), those who did not commonly indicated they noticed a change in breast shape, sensory changes, red or inverted nipple. Similar findings have been observed in other cohorts where the presence of a symptom other than a breast lump increases the risk of later presentation [7,8]. It is suggested that women with “non-lump symptoms” are less likely to attribute that symptom to breast cancer and more likely to delay presentation [7,39]. Further, our finding that women who did not discuss their breast cancer symptom(s) with someone other than their doctor were more likely to delay presentation, is also in keeping with other studies [7,27,46]. Burgess et al. found the likelihood a woman would discuss her concerns was associated with whether they lived with a partner [7]. Similarly, in our study, we found significantly more women who were married or living as married discussed their concerns compared to those without partners.

We found the most common reason women identified for delayed presentation was a lack of awareness of breast cancer symptoms (48%). This is similar to that observed in other studies where over 40% of women who delayed consulting a medical practitioner thought the symptom would go away or was not serious [43]. Current recommendations from health authorities advise that women should be aware of changes such as presence of a breast lump; change in breast size or shape; changes to nipple (including discharge, redness); change in skin or unusual pain [50]. The results in our study indicate a need to continue educating women on the importance of timely medical consultation and an awareness of all potential breast cancer symptoms.

*System-related interval:* While geographic location and area-level disadvantage were not associated with a longer patient-related interval, these factors were strongly associated with intervals of more than 30 days from first medical consultation to diagnosis. Compared to women in major city areas, those living in outer regional and in areas of disadvantage were some 40%–60% more likely to experience a longer SI. This is consistent with other studies, for example, a small study conducted in Iowa, USA found rural women were less likely to receive adequate follow-up diagnostic services, including longer time intervals [51], and Crispo and colleagues who defined system (medical) delay as the time from first physical examination to hospital admittance, found women who attended facilities outside specialist breast health services (mainly located within urban areas) were more likely to experience delays in diagnosis [26].

Of the other sociodemographic factors, we found no independent association with age, education or marital status, but did find that women in full-time employment were more likely to experience a longer system-related interval. While we were unable to find other studies reporting a similar association between employment status and system delay, some studies have reported work and family commitments are factors associated with patient delay [8,44]. It is possible work commitments may hinder making (and keeping) appointments for diagnostic procedures.

Difficulties in access to procedures such as mammography and diagnostic biopsy has been identified as barriers to timely diagnosis in previous studies [52]. In our cohort, women in regional and remote/very remote areas commonly cited difficulties in obtaining appointments in primary care, as well as coordinating appointments for diagnostic procedures. In particular, women in outer regional and remote/very remote areas indicated delays occurred from the initial diagnostic procedures (most commonly clinical breast examination and then mammogram) to diagnostic biopsy (fine needle aspiration or core biopsy). One of the likely reasons for this differential is the limited availability of experienced personnel able to conduct these procedures outside major diagnostic facilities [53]. In a study among rural patients in Western Australia, researchers identified difficulties in timely access to ultrasound-guided breast biopsy as this procedure was not readily available locally [54]. Further, a qualitative study amongst GPs in rural Australia, found one of the emerging themes related to difficulties in coordinating diagnostic procedures for patients who require extensive travel, and GPs additionally felt current guidelines for managing the diagnostic pathway do not take into account the rural practice setting [55].

Improving GP access to regional and rural diagnostic services and timely access to relevant diagnostic procedures would likely result in a reduction in the disparity in system-related intervals between rural and urban breast cancer patients over time. A number of countries have introduced recommended pathways for referral and diagnosis [56,57,58,59]. Evaluation of the effectivness of such pathways has been conducted in Denmark with findings indicating a reduction in the interval between initial GP consultation and diagnosis, with the greatest improvement observed for breast cancer [60]. However, as the evaluation did not examine rural and urban patients separately, it is difficult to know whether such a model would be effective in the Australian setting.

In Australia, while GPs have access to guidelines to assist them in investigating a new breast symptom, these do not include a recommended time frame for investigations to be completed [18]. We are aware of only one other small case series conducted in regional Australia that examined the various components of the diagnostic pathway and found the median interval from symptom awareness to GP consultation was 55 days and from GP consultation to definitive diagnosis was 20 days [61].

*Diagnostic interval*: We did find some women were less likely to experience longer diagnostic intervals. The factors associated with less likelihood of a longer DI mostly related to the clinical characteristics of the breast cancer. For example, women with high grade tumours were nearly 50% less likely to experience a longer DI, with this suggesting a rapidly growing breast lump most likely prompts patients to consult their doctor quickly. Similar results have been observed in other studies. For example, Redondo and colleagues found diagnostic delays of less than 30 days were significantly associated with increased tumour size and poorly differentiated tumours [62].

### 4.2. Screen-Detected Breast Cancer

Approximately 80% of participants in our study received their mammogram results within 14 days. BreastScreen Australia standards state that 90% of all women screened should receive their results within 14 days [20]. Receipt of results varied significantly by residential location with only 60% of women in outer regional and remote locations receiving results within 14 days compared to 90% of women in major city areas (independent of tumour size). We additionally found shorter time intervals in notification of results for women whose mammogram was conducted at a private facility, with the majority of those women being notified within two days. While we are not aware of any other studies conducted in Australia reporting on time intervals from mammogram to notification of results within private screening facilities, our results are in agreement with studies using United States of America (USA) Health Maintenance Organization (HMO) populations, where mammograms are interpreted by radiologists the next working day and patients notified within 24 to 48 hours after issuing of the report [63].

In our study median time from abnormal mammogram to diagnosis was 14 days with over 80% of participants diagnosed within 30 days and 90% within six weeks. Our results are similar to that of several large USA and Canadian studies reporting median times between 12 and 16 days with approximately 80% of women diagnosed within 30 days [63,64,65,66]. BreastScreen Australia recommends women with a screen-detected abnormality should be assessed within 28 days, with their most recent evaluation report indicating around 20% of women were not fully assessed within that time frame [67], similar to our study results.

The strongest factors associated with a diagnostic interval of more than 30 days were rural location and having the mammogram conducted within the BreastScreen program. While there are few studies comparing the diagnostic intervals of screen-detected breast cancer amongst urban and rural women, a study conducted in New Mexico, USA also found longer times to diagnosis for rural women. That study also reported that the decline in time to diagnosis over time for women located in urban areas was not observed for women in rural areas [68]. The authors suggested this trend may have been due to a decreasing use of excisional biopsies and increasing use of “on-site” core biopsies by radiologists in urban areas, as has been reported elsewhere [69,70]. This may be the case in our study, as compared to women from urban areas, those living in rural locations were significantly less likely to have a core biopsy conducted at the same time as their screening mammogram and/or ultrasound.

We found Indigenous women whose cancer was screen-detected were more likely to experience a diagnostic interval of more than 30 days compared to non-Indigenous women. Other studies have reported ethnic disparities associated with diagnostic delay [71,72]. In Australia, Indigenous women have lower rates of breast screening [13], more extensive disease at diagnosis [73], and less optimal clinical management [74]. While we are unable to assess the exact reasons for the longer diagnostic interval for Indigenous women in our study, it is possible that the differences are caused by issues with accessibility and potentially a lack of culturally sensitive and appropriate health care. Providing practical assistance and support for Indigenous women and health care professionals who care for Indigenous women undergoing diagnostic procedures may help reduce this disparity.

Our results highlight differences in diagnostic intervals for those attending public or private screening facilities. We found the median time from abnormal mammogram to diagnosis more than doubled for women attending BreastScreen compared with those attending a private facility. As expected, in our study we found women were more likely to attend private breast screening facilities in major cities, however the association between a longer diagnostic interval and public facilities remained after adjusting for residential location. While comparisons with other studies is difficult, one large USA study observed that facilities with a larger volume of patients (hospital-based screening facilities) were more likely to have long follow-up times with shorter times observed in HMO facilities [64]. Identifying methods to collect and assess screening rates and outcomes of screening among women accessing private breast screening clinics needs to be explored.

### 4.3. Strengths and Limitations

Strengths of our study are that we were able to include a number of individual socio-economic measures (such as employment, marital status, education) as well as screening behaviours (such as frequency of pre-diagnostic mammography and breast self-examination), thus potentially limiting confounding due to patient factors and behaviours. Further, this large cohort of women was ascertained through a population-based cancer registry, which may strengthen the potential generalizability of the findings. We used a structured interview conducted by experienced health interviewers. The clinical characteristics, including date of diagnosis were obtained through the cancer registry or from medical records.

Like other studies [34], the retrospective nature of collecting information about dates of symptom recognition, breast screening, medical consultations and procedures is a limitation of our study. We attempted to improve the accuracy of recall of events through the use of calendars, interviewer probing and quantified the level of accuracy by extracting relevant information from medical records for a random sample of participants. For this group we found reasonable accuracy of recall of events, particularly in relation to the timing of mammography and medical appointments, and this was similar across the covariates we used in our analysis. Thus we believe any recall bias is unlikely to impact on the conclusions of our study. We could not, however, accurately verify the timing of symptom recognition for women whose breast cancer was symptom-detected, rather we relied on the recall of each women. Larsen et al. have previously reported moderate agreement between patients and GPs in relation to the date of symptom recognition and good agreement for the date of initial consultation [75]. While there is likely to be some inaccuracies in the reporting of dates in our study, it is doubtful there would be any differential bias in the recall of dates of events according to residential location or other covariates.

## 5. Conclusions

Our findings indicate significant disparities in the time to diagnosis for both symptom- and screen-detected breast cancer, with women in regional and remote locations more likely to experience diagnostic delay. For some women, a lack of awareness of the importance of potential symptoms of breast cancer resulted in delays in consulting with their medical practitioner. Thus, while in general there is a need to continue efforts to raise awareness of the symptoms of breast cancer and the importance of consulting a medical practitioner in a timely manner, approaches targeting those most at risk of delays in diagnosis should be a priority for public health.

Rural inequality in cancer outcomes in Australia and elsewhere has been evident for a number of decades now. Identifying the barriers that drive these inequalities is paramount. This study has identified some important factors, in particular the difficulty of regional and remote women accessing primary care. The challenge now is in designing and implementing an efficient and effective model of care to minimize avoidable delays and to ensure that all women are diagnosed as quickly as possible.

## Figures and Tables

**Table 1:** Relationship between diagnostic interval and sociodemographic and clinical variables amongst 1560 women in Queensland, Australia with symptom-detected breast cancer: results of logistic regression model.

Variable	% Interval >60 Days	*Crude* *p*-Value	Diagnostic Interval ≤60 Days vs. >60 Days		
			Adjusted Odds Ratio ^1^	95% CI	*p*-Value
Age group		0.53			0.41
<50 years (*n* = 624)	27.4		1.12	0.86, 1.46	
50–79 years (*n* = 936)	26.0		ref		
Indigenous status		0.006			0.005
Non-indigenous (*n* = 1534)	26.1		ref	1.40, 6.98	
Indigenous (*n* = 26)	50.0		3.12		
Residential location		0.003			0.003
Major city (*n* = 854)	23.4		ref		
Inner regional (*n* = 388)	28.4		1.28	0.96, 1.69	
Outer regional (*n* = 263)	30.8		1.50	1.09, 2.06	
Remote/very remote (*n* = 55)	41.8		2.46	1.39, 4.38	
Area-level disadvantage		0.10			
Least disadvantaged (*n* = 658)	23.9				
Middle socioeconomic status (*n* = 336)	27.4		-	-	-
Most disadvantaged (*n* = 566)	29.2				
Marital status		0.70	-		
Married/living as married (*n* = 1142)	26.8		-	-	-
Not married/not living as married (*n* = 418)	25.8				
Education		0.77			
High school or less (*n* = 597)	26.3				
Certificate/vocational (*n* = 346)	28.0		-	-	-
Diploma/Batchelors or higher (*n* = 617)	25.9				
Employment status		0.07			0.10
Full-time (*n* = 559)	32.0		1.45	1.03, 2.06	
Part-time/casual (*n* = 458)	26.0		1.32	0.92, 1.89	
Home duties/not working (*n* = 178)	28.1		1.44	1.01, 2.04	
Retired (*n* = 365)	22.2		ref		
Private health insurance		0.30			
Full (*n* = 935)	25.8				
Some (*n* = 158)	31.7		-	-	-
None (*n* = 467)	26.3				
Frequency of mammogram		0.25			
At least once every 2 years (*n* = 795)	25.3		-	-	-
Irregular/never (*n* = 765)	27.8				
Presence of breast lump		<0.001			<0.001
Yes (*n* = 1121)	22.7		ref		
No (*n* = 439)	36.4		1.84	1.43, 2.36	
Discussed symptoms with someone **^2^**		0.05			0.06
Yes (*n* = 1056)	25.0		ref		
No (*n* = 504)	29.8		1.27	0.99, 1.63	
Tumour size		0.03			0.68
<1.0 cm (*n* = 186)	31.2		ref		
≥1.0 cm to <2 cm (*n* = 516)	24.4		0.82	0.56, 1.21	
≥2.0 cm (*n* = 804)	25.9		0.95	0.65, 1.38	
Unknown (*n* = 54)	40.7		1.00	0.47, 2.11	
Histological grade		<0.001			<0.001
Low (*n* = 175)	34.3		ref		
Intermediate (*n* = 676)	29.1		0.77	0.53, 1.11	
High (*n* = 676)	20.3		0.49	0.33, 0.73	
Unknown (*n* = 33)	60.6		2.58	1.06, 6.27	

**^1^** Logistic model adjusted for age group, Indigenous status, residential location, pre-diagnosis employment, whether presenting symptom included breast lump; **^2^** participant discussed symptoms with someone other than a health professional; dashes indicate variable not included in final model.

**Table 2:** Multivariable logistic regression model showing factors associated with patient-related interval for 1560 women with symptom-detected breast cancer.

Variable	% Interval >30 Days	*Crude* *p*-Value	Patient-Related Interval ≤30 Days vs. >30 Days		
			Adjusted Odds Ratio ^1^	95% CI	*p*-Value
Age group		0.37			0.6
20–49 years (*n* = 624)	29.6		1.07	0.82, 1.41	
50–79 years (*n* = 936)	27.6		ref		
Area-level disadvantage		0.14			0.12
Least disadvantaged (*n* = 658)	27.2		ref		
Middle SES (*n* = 336)	32.7		1.34	1.00, 1.79	
Most disadvantaged (*n* = 566)	27.2		1.03	0.79, 1.34	
Employment status		0.08			0.15
Full-time (*n* = 559)	32.0		1.43	1.02, 2.00	
Part-time/casual (*n* = 458)	26.9		1.13	0.79, 1.61	
Home duties/not working (*n* = 178)	28.6		1.20	0.78, 1.85	
Retired (*n* = 365)	24.7		ref		
Frequency of mammogram		0.005			0.02
At least once every 2 years (*n* = 795)	25.3		ref		
Irregular/never (*n* = 765)	31.6		1.35	1.06, 1.73	
Presence of breast lump		<0.001			<0.001
Yes (*n* = 1121)	24.4		ref		
No (*n* = 439)	38.5		1.88	1.47, 2.39	
Discussed symptoms with someone **^2^**		0.001			0.002
Yes (*n* = 1056)	25.8		ref		
No (*n* = 504)	33.9		1.48	1.16, 1.88	
Histological grade		0.002			0.007
Low (*n* = 175)	35.4		ref		
Intermediate (*n* = 676)	29.1		0.72	0.50, 1.04	
High (*n* = 676)	24.9		0.61	0.42, 0.87	
Unknown (*n* = 33)	48.5		1.56	0.72, 3.37	

**^1^** Logistic model adjusted for all other variables shown in the table; **^2^** Participant discussed breast cancer symptoms with someone other than a health professional.

**Table 3:** Multivariable logistic regression model showing factors associated with system-related interval for 1560 women with symptom-detected breast cancer.

Variable	% Interval >30 Days	*Crude* *p*-Value	System-Related Interval ≤30 Days vs. >30 Days		
			Adjusted Odds Ratio ^1^	95% CI	*p*-Value
Age group		0.16			0.15
20–49 years (*n* = 624)	17.3		1.26	0.91, 1.74	
50–79 years (*n* = 936)	14.6		ref		
Residential location		0.05			0.01
Major city (*n* = 854)	13.8		ref		
Inner regional (*n* = 388)	16.2		1.20	0.84, 1.70	
Outer regional (*n* = 263)	19.8		1.61	1.10, 2.35	
Remote/very remote (*n* = 55)	21.8		1.85	1.00, 3.66	
Area-level disadvantage		0.003			0.008
Least disadvantaged (*n* = 658)	12.9		ref		
Middle SES (*n* = 336)	14.3		1.21	0.81, 1.80	
Most disadvantaged (*n* = 566)	19.8		1.42	1.02, 2.12	
Employment status		<0.001			0.002
Full-time (*n* = 559)	25.8		2.87	1.72, 4.81	
Part-time/casual (*n* = 458)	15.4		1.56	1.00, 2.45	
Home duties/not working (*n* = 178)	15.7		1.61	1.01, 2.55	
Retired (*n* = 365)	11.2		ref		
Presence of breast lump		<0.001			<0.001
Yes (*n* = 1121)	12.6		ref		
No (*n* = 439)	23.7		2	1.49, 2.70	
Tumour size		0.006			0.07
<1.0 cm (*n* = 186)	23.1		ref		
≥1.0 cm to <2.0 cm (*n* = 516)	14.3			0.40, 0.97	
≥2.0 cm (*n* = 804)	14.3			0.44, 1.04	
Unknown (*n* = 54)	24.1			0.13, 0.92	
Histological grade		<0.001			<0.001
Low (*n* = 175)	18.9		ref		
Intermediate (*n* = 676)	19.4		1.06	0.68, 1.66	
High (*n* = 676)	9.4		0.48	0.30, 0.79	
Unknown (*n* = 33)	51.5		7.11	2.61, 19.36	

**^1^** Logistic model adjusted for all other variables shown in the table.

**Table 4:** Factors associated with delays in receiving mammogram results in 1642 women with screen-detected breast cancer: results of logistic regression model.

Variable	% >14 Days	*Crude* *p*-Value	Received Mammogram Results ≤14 Days vs. >14 Days		
			Adjusted Odds Ratio ^1^	95% CI	*p*-Value
Age		0.65			0.2
<50 years (*n* = 239)	18.4		1.29	0.87,1.90	
50–79 years (*n* = 1403)	19.7		ref		
Residential location		<0.001			<0.001
Major city (*n* = 910)	11.0		ref		
Inner regional (*n* = 380)	20.5		1.71	1.20, 2.44	
Outer regional (*n* = 291)	40.6		4.17	2.95, 5.91	
Remote/very remote (*n* = 61)	39.3		3.62	2.00, 6.54	
Area-level disadvantage		<0.001			0.07
Least disadvantaged (*n* = 707)	12.3		ref		
Middle socioeconomic status (*n* = 329)	19.2		1.38	0.94, 2.02	
Most disadvantaged (*n* = 606)	28.1		1.45	1.04, 2.01	
Type of screening facility		<0.001			<0.001
BreastScreen (*n* = 1233)	25.0		9.51	5.21, 17.36	
Private (*n* = 409)	2.9		ref		
Tumour size		0.06			0.01
<1.0 cm (*n* = 625)	21.1		ref		
≥1.0 cm to <2 cm (*n* = 694)	18.6		0.66	0.49, 0.88	
≥2.0 cm (*n* = 316)	18.0		0.63	0.44, 0.92	
Missing (*n* = 7)	28.6		3.15	0.41, 24.01	

**^1^** Logistic model adjusted for all other variables shown in the table.

**Table 5:** Relationship between diagnostic delay and sociodemographic and clinical variables amongst 1642 women in Queensland, Australia with screen-detected breast cancer.

Variable	% Interval >30 Days	*Crude* *p-*Value	Diagnostic Interval ≤30 Days vs. >30 Days		
			Adjusted Odds Ratio ^1^	95% CI	*p*-Value
Age		0.68			0.43
20–49 years (*n* = 239)	15.5		0.84	0.54, 1.30	
50–79 years (*n* = 1403)	16.5		ref		
Indigenous status		0.02			0.04
Non-indigenous (*n* = 1618)	16.1		ref		
Indigenous (*n* = 24)	33.3		2.36	1.03, 5.80	
Residential location		<0.001			<0.001
Major city (*n* = 910)	10.1		ref		
Inner regional (*n* = 380)	16.6		1.41	0.97, 2.04	
Outer regional (*n* = 291)	33.0		3.32	2.31, 4.75	
Remote/very remote (*n* = 61)	29.5		2.35	1.24, 4.44	
Area-level disadvantage		<0.001			0.002
Least disadvantaged (*n* = 707)	9.5		ref		
Middle socioeconomic status (*n* = 329)	18.8		1.58	1.03, 2.56	
Most disadvantaged (*n* = 606)	23.1		1.69	1.17, 2.43	
Marital status		0.81			
Married/living as married (*n* = 1217)	16.5				
Not married/not living as married (*n* = 425)	16.0		-	-	-
Education		0.28			
High school or less (*n* = 648)	17.4				
Certificate/vocational (*n* = 424)	17.5		-	-	-
Diploma/Bachelor’s or higher (*n* = 570)	14.4				
Employment status		0.04			0.03
Full-time (*n* = 438)	23.1		1.70	1.13, 2.44	
Part-time/casual (*n* = 435)	17.8		1.64	1.02, 2.63	
Home duties/not working (*n* = 160)	16.6		1.39	0.96, 2.02	
Retired (*n* = 609)	13.5		ref		
Private health insurance		0.007			0.32
Full (*n* = 1097)	14.6		ref		
Some (*n* = 174)	16.7		1.00	0.63, 1.60	
None (*n* = 371)	21.6		1.28	0.92, 1.78	
Frequency of mammography		0.07			
At least once every 2 years (*n* = 1412)	15.7		-	-	-
Irregular/never (*n* = 230)	20.4				
Type of screening facility		<0.001			<0.001
BreastScreen (*n* = 1233)	19.2		2.10	1.40, 3.17	
Private (*n* = 409)	7.8		ref		
Tumour size		0.009			
<1.0 cm (*n* = 625)	19.5				
≥1.0 cm to <2 cm (*n* = 694)	14.6		-	-	
≥2.0 cm (*n* = 316)	13.6				
Missing (*n* = 7)	42.9				
Histological grade		0.03			
Low (*n* = 419)	19.8				
Intermediate (*n* = 848)	15.6		-	-	
High (*n* = 368)	13.9				
Unknown (*n* = 7)	42.9			-	
Lymph nodes involved		<0.001			<0.001
No (*n* = 1246)	15.6		ref		
Yes (*n* = 369)	16.3		0.98	0.71, 1.37	
Unknown (*n* = 27)	51.8		6.68	2.90, 15.39	

**^1^** Logistic regression model adjusted for age group, Indigenous status, residential location, private health insurance, pre-diagnosis employment, type of screening facility, tumour size and grade, and nodal status; dashes indicate variable not included in final model.

## Abbreviations

The following abbreviations are used in this manuscript:

BC	Breast Cancer
GP	General Practitioner
BCOS	Breast Cancer Outcomes Study
IRSD	Index of Relative Socioeconomic Disadvantage
ABS	Australian Bureau of Statistics
DI	Diagnostic Interval
PI	Patient-related Interval
SI	System-related Interval
LR	Likelihood Ratio Test
OR	Odds Ratios
CI	Confidence Intervals
USA	United States of America
HMO	Health Maintenance Organization
